# Milk, Fresh from the Cow

**Published:** 1908-09-19

**Authors:** J. Howard-Jones

**Affiliations:** Health Department, Town Hall, Newport, Mon.


### Editor's Letter-Box.

**Our correspondents are reminded that prolixity is a great bar to publication, and that brevity of style and conciseness of statement greatly facilitate early insertion.**

### MILK, FRESH FROM THE COW.

*(To the Editor of* The Hospital.*)*

Dear Sir,—The public milk supply of this country is improving at a desperately slow pace, in spite of the fact that the requirements suggested in the paper on "Milk, Fresh from the Cow," in The Hospital, can be carried out without detriment to the cows, and certainly to the advantage of the public. Last month I visited the Home Farm of the Painswick Sanatorium, near the Birdlip; and was delighted to find that the work was conducted on thoroughly hygienic principles, and that the grooming of the cows and washing of the teats was practised systematically, and, in spite of these dangerous (?) practices they were able to take many prizes for butter in the leading dairy shows of the country. The Royal Sanitary Institute have taken the matter up, and will, I understand, communicate with the committees of agricultural shows with the view of getting them to insist on scientific cleanliness at competitions. Yours faithfully,

J. Howard-Jones, M.D., D.Sc.

Health Department, Town Hall, Newport, Mon.
September 14, 1908.

